# A Brief Review on the Influence of Ionic Liquids on the Mechanical, Thermal, and Chemical Properties of Biodegradable Polymer Composites

**DOI:** 10.3390/polym13162597

**Published:** 2021-08-05

**Authors:** Ahmad Adlie Shamsuri, Siti Nurul Ain Md. Jamil, Khalina Abdan

**Affiliations:** 1 Laboratory of Biocomposite Technology, Institute of Tropical Forestry and Forest Products, Universiti Putra Malaysia, UPM Serdang 43400, Selangor, Malaysia; 2 Department of Chemistry, Faculty of Science, Universiti Putra Malaysia, UPM Serdang 43400, Selangor, Malaysia; ctnurulain@upm.edu.my; 3 Centre of Foundation Studies for Agricultural Science, Universiti Putra Malaysia, UPM Serdang 43400, Selangor, Malaysia

**Keywords:** ionic liquid, biodegradable polymer, polymer composites, mechanical, thermal, chemical

## Abstract

Biodegradable polymers are an exceptional class of polymers that can be decomposed by bacteria. They have received significant interest from researchers in several fields. Besides this, biodegradable polymers can also be incorporated with fillers to fabricate biodegradable polymer composites. Recently, a variety of ionic liquids have also been applied in the fabrication of the polymer composites. In this brief review, two types of fillers that are utilized for the fabrication of biodegradable polymer composites, specifically organic fillers and inorganic fillers, are described. Three types of synthetic biodegradable polymers that are commonly used in biodegradable polymer composites, namely polylactic acid (PLA), polybutylene succinate (PBS), and polycaprolactone (PCL), are reviewed as well. Additionally, the influence of two types of ionic liquid, namely alkylimidazolium- and alkylphosphonium-based ionic liquids, on the mechanical, thermal, and chemical properties of the polymer composites, is also briefly reviewed. This review may be beneficial in providing insights into polymer composite investigators by enhancing the properties of biodegradable polymer composites via the employment of ionic liquids.

## 1. Introduction

**Figure 1:**
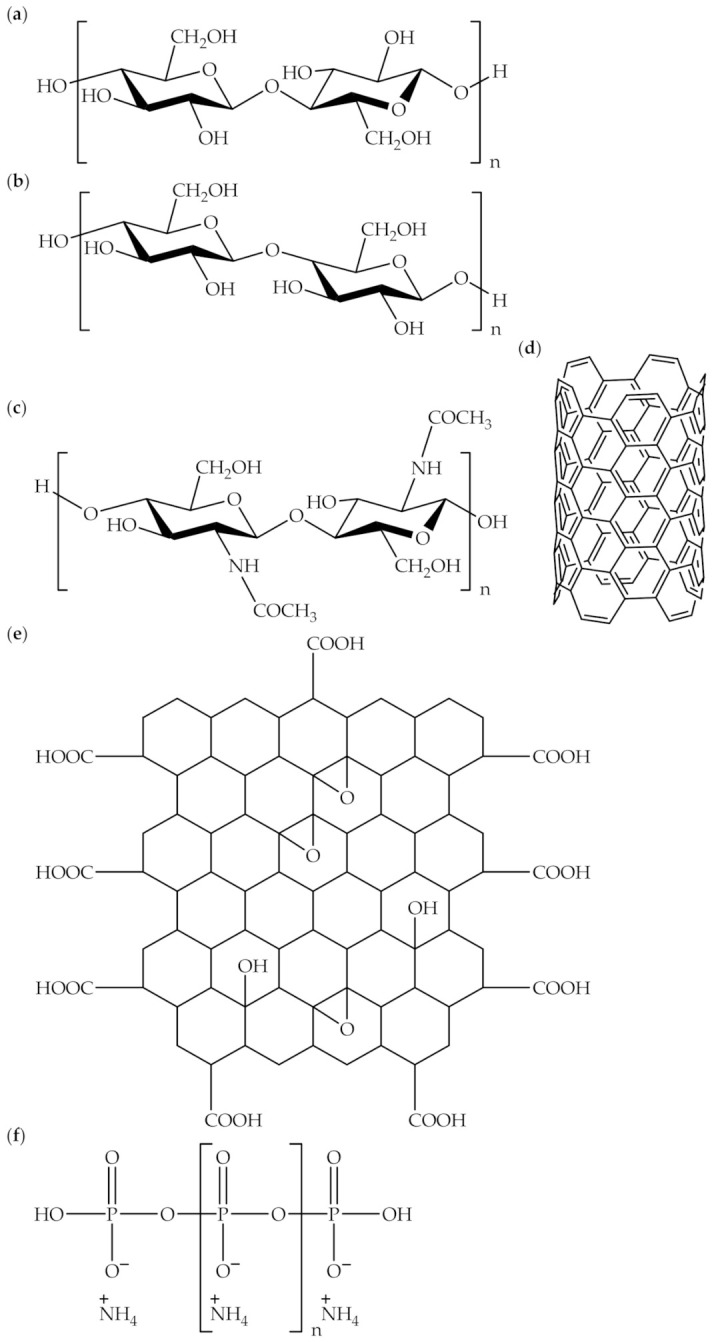
Chemical structures of (**a**) cellulose, (**b**) starch, (**c**) chitin, (**d**) multiwalled carbon nanotubes, (**e**) graphene oxide, and (**f**) ammonium polyphosphate.

In recent years, the consumption of biodegradable polymers in polymer composite technology has expanded, due to the environmental concerns and expansion of the sustainable, biodegradable polymer industry. Biodegradable polymers are a unique class of polymer that decomposes after usage by the bacterial degradation process. They are also both naturally and synthetically manufactured [1]. Synthetic biodegradable polymers are commonly synthesized through a condensation reaction, ring-opening polymerization, and metal catalysts. Biodegradable polymer composites are biodegradable polymeric materials that incorporate either organic or inorganic fillers. Table 1 displays examples of organic and inorganic fillers, utilized for the preparation of biodegradable polymer composites. Organic materials, for example, cellulose [2,3,4,5,6] corn starch [7,8] modified starch [9] rice starch [10] chitin [11,12] rice husk [13] and wood flour [14] have commonly been used as organic fillers to prepare biodegradable polymer composites. This is due to their biodegradability, renewability, economy, and availability [15]. On the other hand, inorganic materials, such as multiwalled carbon nanotubes [16,17,18,19] graphene oxide [20,21] graphene [22,23] layered double hydroxide [24,25,26,27] montmorillonite [28,29] ammonium polyphosphate [30] and zinc oxide [31] have been used as inorganic fillers. Figure 1 shows the chemical structures of cellulose, starch, chitin, multiwalled carbon nanotubes, graphene oxide, and ammonium polyphosphate.

There are many applications of biodegradable polymer composites; some of them have been employed in food packaging, textile, and tissue engineering applications [32,33]. Moreover, the incorporation of inorganic fillers into biodegradable polymers can influence the mechanical properties and hydrophobicity of the fabricated composites, which are very important for biomedical applications [34]. On the other hand, the fabrication of biodegradable polymer composites using organic fillers can be used for lightweight and microfluidic applications [35]. The biodegradable polymer composites with a high dielectric constant and dielectric loss factors can potentially be developed as conductive substrates or semiconductors for electronic applications [36]. The homogenous biodegradable polymer composites fabrication can also be utilized for 3D-printing applications [37]. In addition, biodegradable polymer composites can be fabricated by incorporating bioactive compounds for application in both cosmetic patches and medical patches [38]. The utilization of biodegradable polymer composites is a promising solution for controlled release fertilizer in agriculture applications [39]. Additionally, the in vitro bioactivity of the biodegradable polymer composites is potentially useful for load-bearing orthopedic applications [40].

The employment of ionic liquids in the fabrication of biodegradable polymer composites is a favorable approach. Ionic liquids are molten salts that normally have a low melting point (below 100 °C). They are also non-volatile because they have a very low vapor pressure. Ionic liquids are regarded as an environmentally benign solvent because they can be recycled. Moreover, ionic liquids have attractive solvent properties, for instance, high thermal stability, high polarity, non-flammability, and good electrical conductivity [41]. They also have good solubility with many organic solvents, as well as being able to dissolve most organic materials, including biopolymers and some inorganic materials. In addition, ionic liquids can be customized as required by researchers. Table 2 provides examples of ionic liquids employed in the fabrication of biodegradable polymer composites. It can be seen that most ionic liquids are based on imidazolium and phosphonium cations with distinct counter anions; thus, in this brief review, they have been categorized into two types, specifically alkylimidazolium- and alkylphosphonium-based ionic liquids. This categorization was created because the ionic liquids have different precursors, even though they are subjected to almost the same synthesis procedures.

In the last ten years, various materials have been applied in the fabrication of biodegradable polymer composites with the intention of enhancing the mechanical, thermal, and chemical properties of the polymer composites. The employment of ionic liquids as transformers can provide an advantage because of their responsive chemical structure, which has both large cations and anions that are weakly coordinated. These ions can interact with organic or inorganic fillers and biodegradable polymers. The existence of intermolecular interactions can have an alteration effect on biodegradable polymer composites, and consequently improve the interfacial link between the filler and the polymer matrix [42]. As far as the authors know, no brief review has ever been made concentrating on the influence of ionic liquids on the mechanical, thermal, and chemical properties of biodegradable polymer composites. This is the aim of the categorized review reported in this paper. In addition, although this review is brief, and not particular, it is nonetheless pertinent to other correlated studies.

## 2. Types of Synthetic Biodegradable Polymers

**Figure 2:**
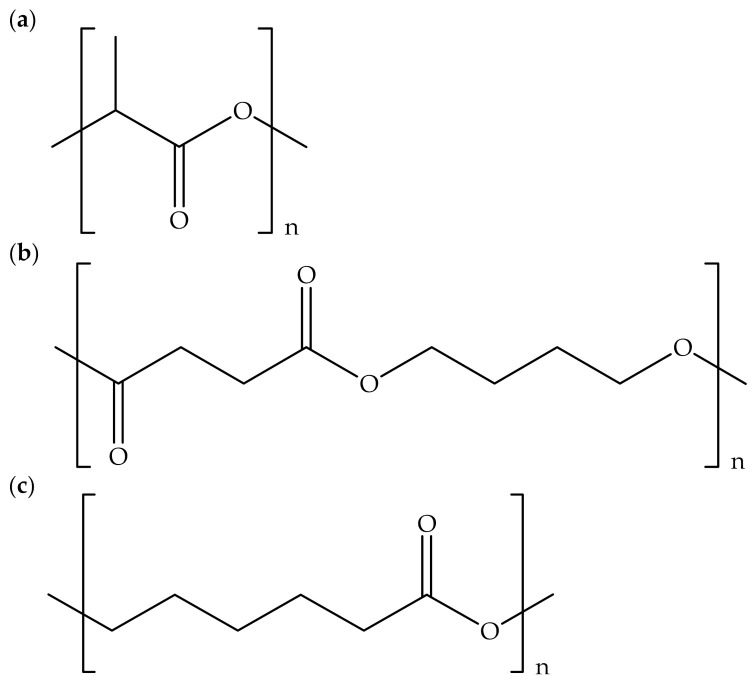
Chemical structures of (**a**) PLA, (**b**) PBS, and (**c**) PCL.

Currently, three types of synthetic biodegradable polymers are frequently used in the fabrication of biodegradable polymer composites, namely polylactic acid (PLA), polybutylene succinate (PBS), and polycaprolactone (PCL). PLA, PBS, and PCL are thermoplastic polyester, synthesized via a condensation reaction. Table 3 shows their characteristics, such as appearance, melting point, solubility, physical, source, and the cost of PLA, PBS, and PCL. It can be observed that PLA has a transparent appearance; meanwhile, PBS and PCL have opaque appearance due to their structures, which do not allow light to pass through them. PLA has a higher melting point compared to PBS and PCL because PLA has a highly crystalline structure. Nevertheless, PBS and PCL are easily soluble in a common organic solvent such as chloroform, while PLA has good solubility in dimethylformamide [6]. Moreover, PBS and PCL have tougher and more flexible properties, respectively, than PLA, which has a brittle character due to its high glass transition temperature. However, PLA can be obtained from bio-based sources, such as sugar cane, sugar beet, corn, and cassava, whereas PBS and PCL are from petrochemical-based sources. Additionally, the cost of PLA is lower compared to PBS and PCL. Figure 2 exhibits the chemical structures of PLA, PBS, and PCL.

The biodegradation process of biodegradable polymers typically involves the digestion of the polymers by microorganisms, followed by their conversion into water and carbon dioxide [43]. Biodegradation of synthetic biodegradable polymers such as PLA, PBS, and PCL depends on their degradation condition, as they biodegrade more quickly in compost than in soil. This is due to the fact that compost contains more microorganisms and natural enzymes that can speed up the polymers’ biodegradation process [44]. The incorporation of inorganic clay into PLA for biodegradable polymer composites fabrication can also improve biodegradation. This is because the presence of the hydrophilic filler can facilitate the diffusion of water in the PLA matrix, which induces hydrolysis [45]. In addition, the biodegradation of polymer composites that consist of a PBS matrix and cotton fiber is higher compared to the neat PBS. This is attributed to the presence of natural fiber in the composites, which increases the biodegradation rate of PBS [46]. Additionally, PCL composites containing a higher starch filler content showed a higher level of biodegradation [47]. Therefore, the selection of proper fillers and contents can considerably affect the biodegradation process of the biodegradable polymer composites in various conditions.

## 3. Types of Ionic Liquids for Biodegradable Polymer Composites

### 3.1. Alkylimidazolium-Based Ionic Liquids

**Figure 3:**
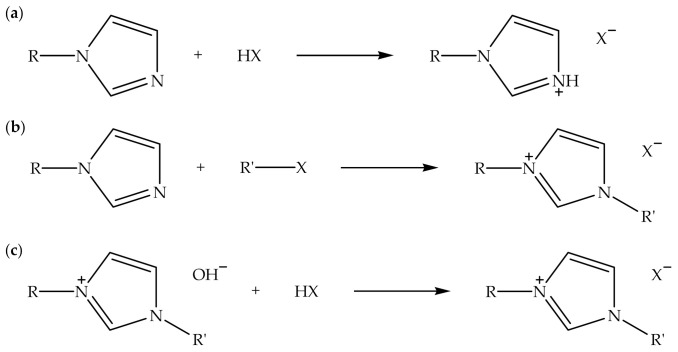
Schematic of the (**a**) protonation, (**b**) alkylation, and (**c**) neutralization reactions for synthesis of alkylimidazolium-based ionic liquids.

Alkylimidazolium-based ionic liquids can be synthesized through protonation, alkylation, or neutralization reactions [48]. Figure 3 shows a schematic of the protonation, alkylation, and neutralization reactions for the synthesis of alkylimidazolium-based ionic liquids. The protonation of *N*-alkylimidazole with hydrohalic acid is usually conducted in a polar solvent, such as ethanol, at slightly above room temperature while stirring. In addition, the alkylation of *N*-alkylimidazole with alkyl halide can be carried out under a reflux condition at an elevated temperature while stirring. The neutralization of alkylimidazolium hydroxide with hydrohalic acid is commonly performed in a polar solvent such as ethanol while stirring at room temperature [48]. Table 4 indicates the types of alkylimidazolium-based ionic liquids, fillers, polymer matrices, and fabrication processes of biodegradable polymer composites. It can be perceived that alkylimidazolium-based ionic liquids with halide counter anions are typically employed in the fabrication of biodegradable polymer composites [2,7,8,9,17,23] compared to other counter anions. This is probably because the employment of the ionic liquids is cost-effective and applicable to many polymer matrices. Moreover, organic fillers are regularly utilized with alkylimidazolium-based ionic liquids, and the fabrication of the polymer composites can be carried out via solution-blending and melt-mixing processes.

### 3.2. Alkylphosphonium-Based Ionic Liquids

**Figure 4:**
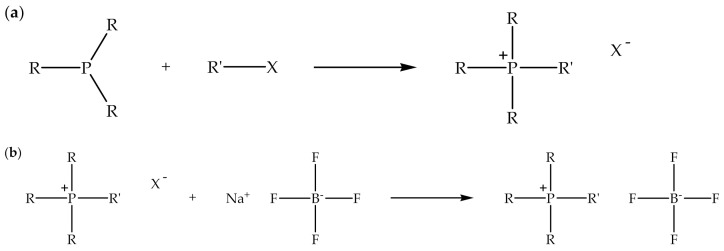
Schematic of the (**a**) alkylation and (**b**) metathesis reactions for synthesis of alkylphosphonium-based ionic liquids.

Alkylphosphonium-based ionic liquids can also be synthesized through an alkylation reaction [49] as alkylimidazolium-based ionic liquids. In addition, similar to the alkylimidazolium-based ionic liquids, alkylphosphonium-based ionic liquids are capable of reacting with alkali salts via metathesis reaction to produce ionic liquids with bulky counter anions [30]. Figure 4 indicates the schematic of the alkylation and metathesis reactions for the synthesis of alkylphosphonium-based ionic liquids. The alkylation of *N*,*N*,*N*-alkylphosphine with alkyl halide is commonly carried out in a non-polar solvent, for example, toluene, at an elevated temperature while stirring [50]. In addition, the metathesis reaction of alkylphosphonium halide with sodium tetrafluoroborate can be performed in a polar solvent, for instance, water, while stirring at slightly above room temperature [30]. Table 5 displays the types of alkylphosphonium-based ionic liquids, fillers, polymer matrices, and fabrication processes of biodegradable polymer composites. It can be observed that alkylphosphonium-based ionic liquids with various types of counter anion can be employed in the fabrication of the polymer composites. Moreover, unlike the alkylimidazolium-based ionic liquids, alkylphosphonium-based ionic liquids are frequently employed with inorganic fillers. Melt-mixing and polymerization are preferable processes for the fabrication of biodegradable polymer composites.

## 4. Influence of Ionic Liquids on the Mechanical, Thermal and Chemical Properties of the Composites

### 4.1. Influence of Alkylimidazolium-Based Ionic Liquids

Table 6 shows the mechanical, thermal, and chemical properties of biodegradable polymer composites influenced by alkylimidazolium-based ionic liquids. The Cel/PLA composite films were fabricated employing [Bmim][OAc] ionic liquid as a solvent [6]. The mechanical, thermal, and chemical properties of the composite films were characterized using universal tensile tester, thermal gravimetric analyzer, differential scanning calorimeter, Fourier transform infrared spectrometer, and an X-ray diffractometer. The mechanical properties, such as the tensile strength and elongation at break of the composite films, increased by up to 52% and 152%, respectively, compared to the pure cellulose film. This was caused by the presence of PLA, which homogeneously hybridized with cellulose and consequently improved the tensile strength as well as the flexibility of the composite films. On the other hand, the thermal properties, such as the decomposition temperature of the composite films, increased by up to 33% in comparison to the pure cellulose film. This was due to the good thermal stability of the composite films [6]. In addition, the chemical properties of the composite films, such as the infrared absorption bands of the O–H stretching vibrations, shifted to higher wavenumber regions compared to the O–H stretching vibration of the neat cellulose. This was attributed to the existence of a hydrogen bonding interaction between the H atoms of the hydroxyl groups in cellulose and the O atoms of the carbonyl groups in PLA, which impaired the hydrogen bonding interaction in the neat cellulose. Moreover, the X-ray diffraction peaks in the composite films disappeared in comparison to the original cellulose and PLA. This was due to the amorphous state existent in the composite films [6]. Hence, it can be inferred that the employment of [Bmim][OAc] ionic liquid gives Cel/PLA composite films high tensile strength and elongation properties, as well as good thermal stability and good interaction between their components.

Meanwhile, CS/PBS composites were fabricated by employing [Bmim][l] ionic liquid as a plasticizer [7]. The mechanical, thermal, and chemical properties of the composites were characterized by means of universal testing machine, thermal gravimetric analyzer, differential scanning calorimeter, Fourier transform infrared spectrometer, and an X-ray diffractometer. The mechanical properties, such as the elongation at break of the composite containing [Bmim][l] increased by up to 323% compared to the CS/PBS composite. This was attributed to the presence of [Bmim][l] which weakened the intermolecular force of CS, caused molecular chains of CS and PBS to be more integrated, and subsequently enhanced the toughness of the composite. Nevertheless, the tensile strength and Young’s modulus of the composite decreased, which was ascribed to the improved compatibility between CS-[Bmim][l] and PBS. Furthermore, the thermal properties, such as the decomposition temperature and glass transition temperature of the composite, decreased, which was caused by the reduction in the thermal stability of the composite containing [Bmim][l] [7]. However, the melting temperature remained almost unchanged for the composite. In addition, the chemical properties, such as the infrared absorption bands of the O–H and C=O stretching vibrations, of the composite containing [Bmim][l] shifted to higher wavenumber regions in comparison to the O–H and C=O stretching vibrations of the CS/PBS composite. This was due to the formation of interactions between the cations of [Bmim][l] and O atoms of CS hydroxyl and PBS carbonyl, as well as between the anions of [Bmim][l] and H atoms of CS hydroxyl. Nonetheless, the X-ray diffraction peaks in the composite decreased, induced by the diminution of the crystallinity of the composite [7]. Therefore, it can be concluded that the employment of [Bmim][I] ionic liquid provides the CS/PBS composite with a high elongation character, and its components have good interactions.

Additionally, the Cel/PLA composites were fabricated by employing [Bmim][BF_4_] ionic liquid as a modifier [3]. The mechanical, thermal, and chemical properties of the composites and their components were characterized using tensile testing machine, thermal gravimetric analyzer, differential scanning calorimeter, and Fourier transform infrared spectrometer. The mechanical properties, such as the tensile strength of the composites containing [Bmim][BF_4_]-modified cellulose, slightly decreased, due to the increase in the toughness of the composites. However, the elongation at break of the composites increased by up to 283% compared to the neat PLA. This was caused by the plasticization effect of the ionic liquid, which enhanced the slippage between molecular chains of PLA, and acted as a plasticizer. However, the Young’s modulus was significantly unchanged for the composites, which correlated with the reinforcement effect of stiff cellulose. On the other hand, the thermal properties, such as the decomposition temperature of the [Bmim][BF_4_]-modified cellulose, increased by up to 211% in comparison to the pristine cellulose. This was attributed to the existence of an ionic interaction between the ionic liquid and cellulose [3]. In addition, the composites showed a melting crystallization peak at a temperature of 90 °C compared to the neat PLA. This was because the incorporation of [Bmim][BF_4_]-modified cellulose considerably improved the crystallization rate of PLA. Furthermore, the chemical properties, such as the infrared absorption bands of the C–H stretching vibrations, of the [Bmim][BF_4_]-modified cellulose, shifted to lower wavenumber regions in comparison to the C–H stretching vibrations of the ionic liquid. This was due to the interaction of Bmim^+^ cations with negatively charged groups of cellulose, which disturbed the hydrogen bonding formation between cellulose [3]. Thus, it can be deduced that the employment of [Bmim][BF_4_] ionic liquid grants Cel/PLA composites with a high elongation character, and high decomposition and melting temperatures, as well as good interaction between their components.

Additionally, RS/PBS composites were fabricated by employing [Dmim][NTf_2_] ionic liquid as a compatibilizer [10]. The mechanical, thermal, and chemical properties of the composites were characterized by means of universal testing machine, thermal gravimetric analyzer, differential scanning calorimeter, and Fourier transform infrared spectrometer. The mechanical properties, such as the elongation at break of the composites containing [Dmim][NTf_2_] increased by up to 233% compared to the RS/PBS composite. This was attributed to the amphiphilic character of [Dmim][NTf_2_] which is capable of interacting with both polar RS and non-polar PBS. Nevertheless, the tensile strength and tensile modulus of the composites marginally decreased, which was caused by the decrease in the stiffness of the composites. On the contrary, the thermal properties, such as the decomposition temperature of the composites containing [Dmim][NTf_2_] increased by up to 2.3% in comparison to the RS/PBS composite. This was ascribed to the presence of [Dmim][NTf_2_], which induced the interactions between each of the components and, as a result, enhanced the thermal stability of the composites [10]. However, the melting temperature of the composites slightly decreased, which was also due to the existence of RS-PBS intermolecular interactions. Moreover, the chemical properties, such as the infrared absorption bands of the O–H stretching and C–O stretching vibrations, of the composites containing [Dmim][NTf_2_] shifted to lower wavenumber regions compared to the RS/PBS composite. This was because of the formation of the ion-dipole force between the polar cations of the ionic liquid and the polar hydroxyl groups of RS. In addition, the infrared absorption bands of the C–H stretching vibrations of the composites containing [Dmim][NTf_2_] shifted to lower wavenumber regions in comparison to the RS/PBS composite. This was due to the non-polar alkyl chain of [Dmim][NTf_2_] interacting with the non-polar group of PBS via hydrophobic-hydrophobic interaction [10]. Hence, it can be inferred that the employment of [Dmim][NTf_2_] ionic liquid gives RS/PBS composites a high elongation character, good thermal stability, and good interactions between their components.

On the other hand, the Chi/PLA composite fibers were fabricated by employing [Emim][OAc] ionic liquid as a solvent [11]. The mechanical, thermal, and chemical properties of the composite fibers were characterized by using the MTS Q-Test 25 instrument, thermal gravimetric analyzer, differential scanning calorimeter, and Fourier transform infrared spectrometer. The mechanical properties, such as tensile strength, elongation at break, and Young’s modulus of the composite fibers, increased by up to 58%, 40%, and 193%, respectively, compared to the neat chitin fiber. This was caused by the presence of PLA, which improved the strength and plasticity properties of the composite fibers. Furthermore, the thermal properties, such as the decomposition temperature of the composite fibers, increased by up to 19% in comparison to the neat chitin fiber. This was due to the homogeneous composite fibers, which depended on the content of PLA. Additionally, the melting temperature of the composite fibers increased by up to 13% compared to the neat PLA. This was attributed to the formation of a second type of crystallite size population with a different level of surface free energy [11]. On top of that, the chemical properties, such as the infrared absorption bands of the C=O stretching vibrations, of the composite fibers, shifted to lower wavenumber regions in comparison to the C=O stretching vibration of the neat PLA. This was due to the existence of hydrogen bonding interactions between the amide groups of chitin and the carbonyl groups of PLA in the composite fibers, which enhanced the strength of the composite fibers [11]. Therefore, it can be concluded that the employment of [Emim][OAc] ionic liquid provides Chi/PLA composite fibers with high tensile strength, elongation, and Young’s modulus properties, as well as high decomposition and melting temperatures, and their components have good interactions.

### 4.2. Influence of Alkylphosphonium-Based Ionic Liquids

Table 7 indicates the mechanical, thermal, and chemical properties of biodegradable polymer composites influenced by alkylphosphonium-based ionic liquids. The MMT/PBS composites were fabricated by employing [P_6,6,6,14_][Cl] ionic liquid as a modifier [28]. The mechanical, thermal, and chemical properties of the composites and their components were characterized by means of universal testing machine, thermal gravimetric analyzer, dynamic mechanical analyzer, and X-ray diffractometer. The mechanical properties, such as the tensile strength of the composite, increased by up to 20% compared to the pure PBS. This was due to the uniform dispersion of [P_6,6,6,14_][Cl]-modified MMT with exfoliation-predominate structures, which confined the segmental motion of PBS macro-molecules. Moreover, the thermal properties, such as the decomposition temperature of the [P_6,6,6,14_][Cl]-modified MMT, decreased, which was due to the low thermal decomposition of P_6,6,6,14_^+^ cations that are intercalated into the MMT interlayers via cation exchange and bound to surface sites of the MMT through electrostatic interaction [28]. In contrast, the glass transition temperature of the composite increased by up to 100% in comparison to the pure PBS. This was caused by the existence of a strong interaction between the [P_6,6,6,14_][Cl]-modified MMT and the PBS matrix, which constrained the movements of the PBS molecular chain segments. On the other hand, the chemical properties, such as the X-ray diffraction peaks in the composite, decreased, which was induced by the enhancement of the distance between the MMT layers, and subsequent enhancement of their exfoliating degree [28]. Thus, it can be deduced that the employment of [P_6,6,6,14_][Cl] ionic liquid provides the MMT/PBS composite with a high tensile strength and high glass transition temperature, and its components have a good interaction.

Meanwhile, the LDH/PCL composite films were fabricated by employing [P_6,6,6,14_][C_9_H_19_CO_2_] ionic liquid as a modifier [26]. The mechanical, thermal, and chemical properties of the composite films and their components were characterized by using a universal testing machine, thermal gravimetric analyzer, differential scanning calorimeter, Fourier transform infrared spectrometer, and X-ray diffractometer. The mechanical properties, such as the tensile strength, elongation at break, and Young’s modulus of the composite film containing [P_6,6,6,14_][C_9_H_19_CO_2_]-modified LDH, increased by up to 44%, 20%, and 2.1%, respectively, compared to the LDH/PCL composite film. This was attributed to the presence of rigid ionic liquid-modified LDH, which was homogeneously dispersed in the PCL matrix. Furthermore, the thermal properties, such as the decomposition temperature of the composite film containing [P_6,6,6,14_][C_9_H_19_CO_2_]-modified LDH increased by up to 4.7% in comparison to the neat PCL film. This was due to the improvement in the thermal stability of the composite film, which correlated with the formation of much larger PCL crystallites in the composite film [26]. In addition, the melting temperature of the composite film increased by up to 6.2% compared to the neat PCL film. This was also caused by the good dispersion of [P_6,6,6,14_][C_9_H_19_CO_2_]-modified LDH. In addition, the chemical properties, such as the infrared absorption bands of the C–H stretching and –(C=O)O– stretching vibrations, were existent in the [P_6,6,6,14_][C_9_H_19_CO_2_]-modified LDH. This confirmed that LDH is successfully modified by the ionic liquid. Moreover, the X-ray diffraction peak of the composite film containing [P_6,6,6,14_][C_9_H_19_CO_2_]-modified LDH increased by up to 30% in comparison to the LDH/PCL composite film. This was induced by the nucleating effect of ionic-liquid-modified LDH on PCL crystallization [26]. Hence, it can be inferred that the employment of [P_6,6,6,14_][C_9_H_19_CO_2_] ionic liquid gives the LDH/PCL composite film a high tensile strength, elongation, and Young’s modulus properties, as well as high decomposition and melting temperatures.

Additionally, the APP/PLA composites were fabricated by employing [P_4,4,4,4_][BF_4_] ionic liquid as a synergist [30]. The mechanical, thermal, and chemical properties of the composites were characterized by means of a universal testing machine, thermal gravimetric analyzer, differential scanning calorimeter, and Fourier transform infrared spectrometer. The mechanical properties, such as the tensile strength, elongation at break, and Young’s modulus of the APP/PLA/[P_4,4,4,4_][BF_4_] composites, increased by up to 4.8%, 1649%, and 7.1%, respectively, compared to the APP/PLA composite. This was ascribed to the presence of [P_4,4,4,4_][BF_4_], which has a significant toughening effect on the APP/PLA composites [30]. Nonetheless, the thermal properties, such as the decomposition temperature of the composites, decreased. This was due to the existence of interaction between [P_4,4,4,4_][BF_4_] and PLA, which promoted the thermal decomposition of PLA. However, the glass transition temperature and melting temperature remained almost unchanged for the APP/PLA/[P_4,4,4,4_][BF_4_] composites. On the other hand, the chemical properties, such as the intensity of the infrared absorption bands of the gas products, of the composites, decreased, which was due to the minor release of gas pyrolysis products [30]. Therefore, it can be concluded that the employment of [P_4,4,4,4_][BF_4_] ionic liquid provides APP/PLA composites with a high tensile strength, elongation, and Young’s modulus properties, as well as their components having a good level of interaction.

The ZnO/PCL composite films were fabricated by employing [P_6,6,6,14_][C_9_H_19_CO_2_] ionic liquid as a modifier [31]. The mechanical, thermal, and chemical properties of the composite films and their components were characterized using a universal testing machine, thermal gravimetric analyzer, differential scanning calorimeter, Fourier transform infrared spectrometer, and X-ray diffractometer. The mechanical properties, such as the tensile strength and elongation at break of the composite films containing [P_6,6,6,14_][C_9_H_19_CO_2_]-modified ZnO, increased by up to 36% and 104%, respectively, compared to the ZnO/PCL composite film. This was caused by the good homogeneity of the ionic liquid-modified ZnO dispersion in the PCL matrix. Furthermore, the Young’s modulus of the composite films containing [P_6,6,6,14_][C_9_H_19_CO_2_]-modified ZnO increased by up to 90% in comparison to the neat PCL film. This was attributed to the high rigidity character, which substantially improved the stiffness of the composite films. In addition, the thermal properties, such as the decomposition temperature, of the composite films containing [P_6,6,6,14_][C_9_H_19_CO_2_]-modified ZnO increased by up to 1.0% compared to the ZnO/PCL composite film. This was because the modification of ZnO with the ionic liquid enhanced the compatibility of ZnO-PCL, and consequently improved the thermal stability of the composite films [31]. In contrast, the glass transition temperature of the composite films slightly decreased, which was ascribed to the plasticizing effect of ionic-liquid-modified ZnO on the PCL matrix. However, the melting temperature remained almost unchanged for the composite films. In addition, the chemical properties, such as the infrared absorption bands of the carboxylate and C–H stretching vibrations, were present in the [P_6,6,6,14_][C_9_H_19_CO_2_]-modified ZnO. This proved that ZnO was positively modified by the ionic liquid. Moreover, the X-ray diffraction peaks of the composite films decreased. This was induced by the exfoliation of ionic liquid-modified ZnO, which provided a good dispersion in the composite films [31]. Thus, it can be deduced that the employment of [P_6,6,6,14_][C_9_H_19_CO_2_] ionic liquid provides ZnO/PCL composite films with high tensile strength, elongation, and Young’s modulus properties, as well as a high decomposition temperature.

## 5. Conclusions

Types of fillers, synthetic biodegradable polymers, ionic liquids, and the fabrication processes of biodegradable polymer composites, were briefly reviewed in this paper. The important properties, for instance, mechanical, thermal and chemical, of the biodegradable polymer composites were also described in this brief review. Ionic liquids employed in the fabrication of biodegradable polymer composites are mostly based on imidazolium and phosphonium cations combined with different counter anions. Alkylimidazolium-based ionic liquids are regularly employed as a solvent, plasticizer, modifier, and compatibilizer for the fabrication of organic filler/synthetic biodegradable polymer composites. Meanwhile, alkylphosphonium-based ionic liquids are frequently employed as a modifier and synergist for the fabrication of inorganic filler/synthetic biodegradable polymer composites. The employment of alkylimidazolium- and alkylphosphonium-based ionic liquids can effectively improve the mechanical, thermal, and chemical properties of biodegradable polymer composites. In addition, the ionic liquids can form intermolecular interactions between organic or inorganic fillers and biodegradable polymer matrices. Alkylimidazolium-based ionic liquids provide biodegradable polymer composites with a high elongation character, good thermal stability, and good compatibility. in addition, alkylphosphonium-based ionic liquids provide biodegradable polymer composites with a high tensile strength, good thermal stability, and good interaction. This brief review may be beneficial not only for polymer composite investigators, but also for the employment of ionic liquids in improving the properties of biodegradable polymer composites.

## Figures and Tables

**Table 1:** Examples of organic and inorganic fillers utilized for preparation of biodegradable polymer composites.

Organic Filler	Abbreviation	Inorganic Filler	Abbreviation
Cellulose	Cel	Multiwalled carbon nanotubes	MWCNTs
Corn starch	CS	Graphene oxide	GO
Modified starch	MS	Graphene	Gra
Rice starch	RS	Layered double hydroxide	LDH
Chitin	Chi	Montmorillonite	MMT
Rice husk	RH	Ammonium polyphosphate	APP
Wood flour	WF	Zinc oxide	ZnO

**Table 2:** Examples of ionic liquids employed in the fabrication of biodegradable polymer composites.

Ionic Liquid	Abbreviation	References
1-Allyl-3-methylimidazolium chloride	[Amim][Cl]	[16,20]
1-Butyl-3-methylimidazolium acetate	[Bmim][OAc]	[6]
1-Butyl-3-methylimidazolium bromide	[Bmim][Br]	[7]
1-Butyl-3-methylimidazolium chloride	[Bmim][Cl]	[2,7,8,9,17,22]
1-Butyl-3-methylimidazolium iodide	[Bmim][I]	[7]
1-Butyl-3-methylimidazolium hexafluoroantimonate	[Bmim][SbF_6_]	[17]
1-Butyl-1-methylpyrrolidinium hexafluorophosphate	[Bmpy][PF_6_]	[21]
1-Butyl-3-methylimidazolium tetrafluoroborate	[Bmim][BF_4_]	[3]
1-Butyl-3-methylimidazolium trifluoromethanesulfonate	[Bmim][OTf]	[17]
1-Carboxymethyl-3-methylimidazolium tetrafluoroborate	[Cmmim][BF_4_]	[18]
1-Dodecyl-3-methylimidazolium bis(trifluoromethylsulfonyl)imide	[Dmim][NTf_2_]	[10]
1,5-Diazabicyclo [4.3.0]non-5-enium acetate	[DBNH][OAc]	[4]
1-((Ethoxycarbonyl)methyl)-3-methylimidazolium bromide	[Ecmmim][Br]	[23]
1-Ethyl-3-methylimidazolium acetate	[Emim][OAc]	[5,11,12,13]
1-Methylimidazolium-3-butylsulfonic acid chloride	[MimbSO_3_H·Cl]	[19]
Trihexyltetradecylphosphonium bistriflamide	[P_6,6,6,14_][TFSA]	[14]
Trihexyltetradecylphosphonium bis(2-ethylhexyl)phosphate	[P_6,6,6,14_][(EH)_2_PO_4_]	[24]
Trihexyltetradecylphosphonium bis(2,4,4-trimethylpentyl)phosphinate	[P_6,6,6,14_][(i-C_8_)PO_2_]	[24,25]
Trihexyltetradecylphosphonium chloride	[P_6,6,6,14_][Cl]	[28]
Trihexyltetradecylphosphonium decanoate	[P_6,6,6,14_][C_9_H_19_CO_2_]	[25,26,31]
Trihexyltetradecylphosphonium 2-ethylhexanoate	[P_6,6,6,14_][Oct]	[24,27]
Trihexyltetradecylphosphonium hexafluorophosphate	[P_6,6,6,14_][PF_6_]	[25]
Tetrabutylphosphonium tetrafluoroborate	[P_4,4,4,4_][BF_4_]	[30]

**Table 3:** Appearance, melting point, solubility, physical, source, and cost of PLA, PBS, and PCL.

Characteristics	PLA	PBS	PCL
Appearance	Transparent	Opaque	Opaque
Melting point	160 °C	115 °C	60 °C
Solubility	Dimethylformamide	Chloroform	Chloroform
Physical	Brittle	Tough	Flexible
Source	Bio-based	Petrochemical	Petrochemical
Cost	Low	High	Moderate

**Table 4:** Types of alkylimidazolium-based ionic liquids, fillers, polymer matrices, and fabrication processes of biodegradable polymer composites.

Alkylimidazolium-Based Ionic Liquid	Filler	Polymer Matrix	Fabrication Process	References
[Bmim][OAc]	Cel	PLA	Solution blending	[6]
[Bmim][Cl]	MWCNTs	PCL	Solution blending	[17]
[Bmim][Cl]	Cel	PCL	Polymerization	[2]
[Bmim][Cl]	CS	PBS	Melt mixing	[8]
[Bmim][Cl]	MS	PBS	Melt mixing	[9]
[Bmim][I]	CS	PBS	Melt mixing	[7]
[Bmim][BF_4_]	Cel	PLA	Melt mixing	[3]
[Dmim][NTf_2_]	RS	PBS	Melt mixing	[10]
[Ecmmim][Br]	Gra	PLA	Solution blending	[23]
[Emim][OAc]	Chi	PLA	Solution blending	[11]
[Emim][OAc]	RH	PLA	Melt mixing	[13]

**Table 5:** Types of alkylphosphonium-based ionic liquids, fillers, polymer matrices, and fabrication processes of biodegradable polymer composites.

Alkylphosphonium-Based Ionic Liquid	Filler	Polymer Matrix	Fabrication Process	References
[P_6,6,6,14_][TFSA]	WF	PLA	Melt mixing	[14]
[P_6,6,6,14_][(EH)_2_PO_4_]	LDH	PCL	Polymerization	[24]
[P_6,6,6,14_][(i-C_8_)PO_2_]	LDH	PLA	Melt mixing	[25]
[P_6,6,6,14_][Cl]	MMT	PBS	Melt mixing	[28]
[P_6,6,6,14_][C_9_H_19_CO_2_]	LDH	PCL	Polymerization	[26]
[P_6,6,6,14_][Oct]	LDH	PCL	Mechanical milling	[27]
[P_6,6,6,14_][PF_6_]	LDH	PLA	Melt mixing	[25]
[P_4,4,4,4_][BF_4_]	APP	PLA	Melt mixing	[30]
[P_6,6,6,14_][C_9_H_19_CO_2_]	ZnO	PCL	Polymerization	[31]

**Table 6:** Mechanical, thermal, and chemical properties of biodegradable polymer composites influenced by alkylimidazolium-based ionic liquids.

Biodegradable Polymer Composite	Alkylimidazolium-Based Ionic Liquid	Mechanical			Thermal			Chemical		References
		TS	EB	YM	*T*_d_	*T*_g_	*T*_m_	FTIR	XRD	
Cel/PLA	[Bmim][OAc]	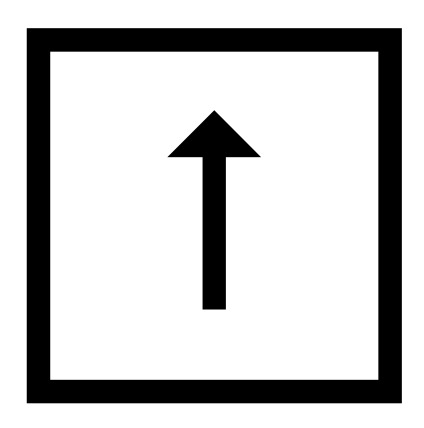	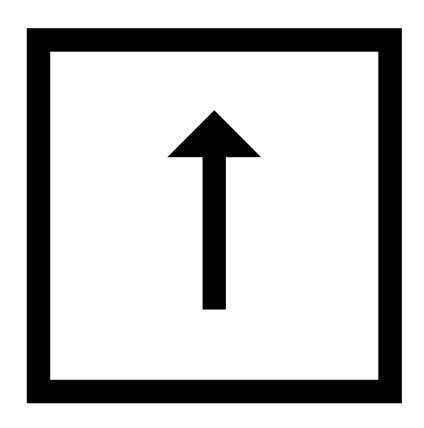	n/a	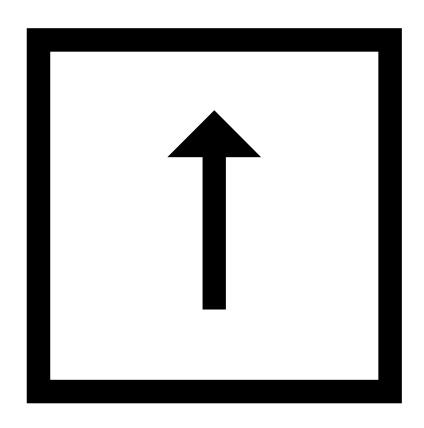	n/a	n/a	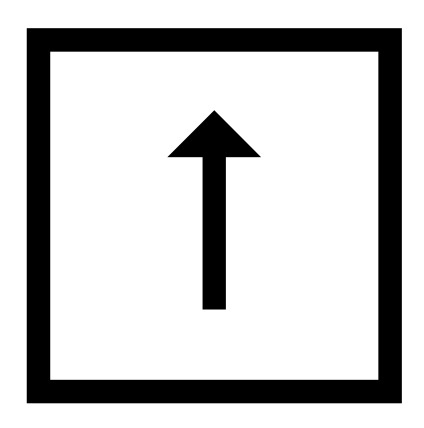	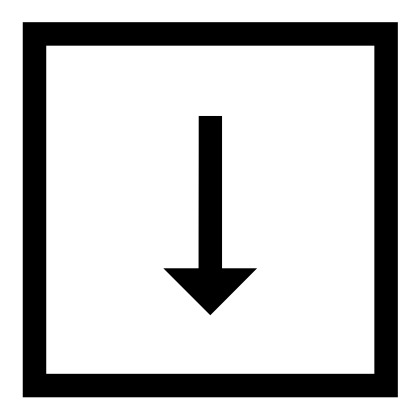	[6]
CS/PBS	[Bmim][I]	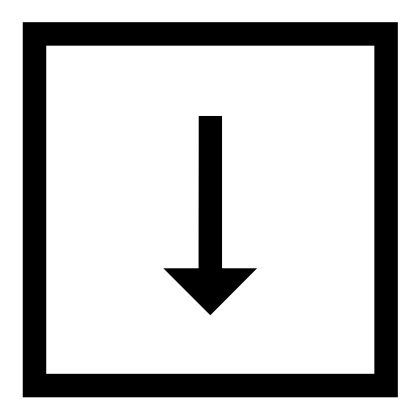	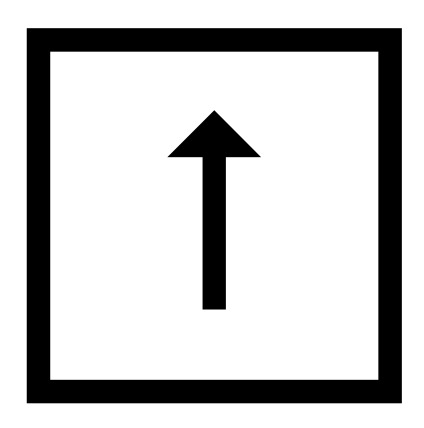	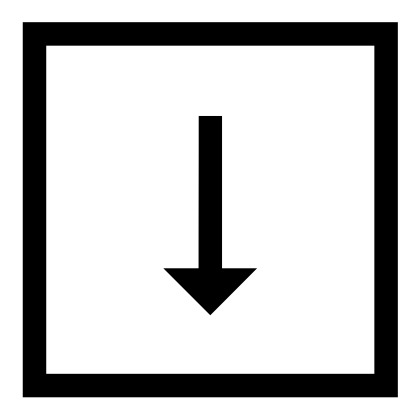	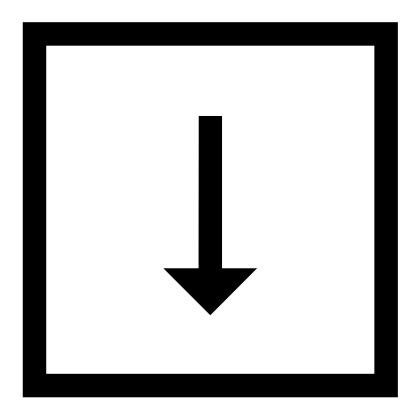	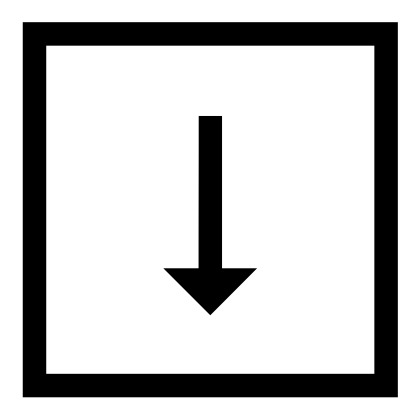	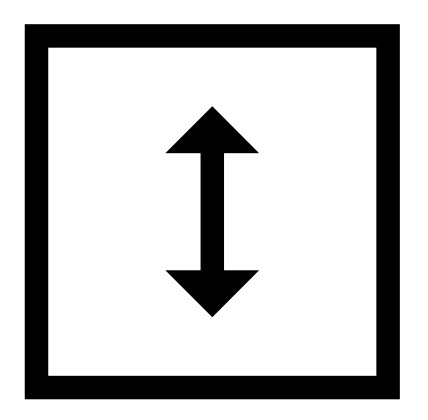	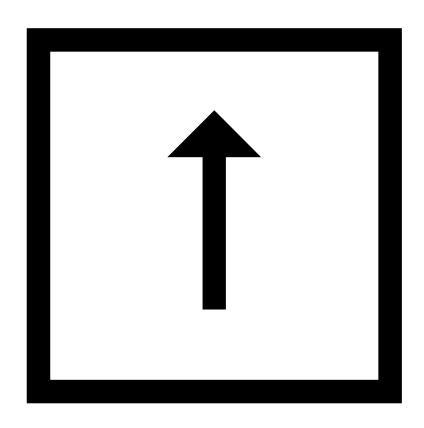	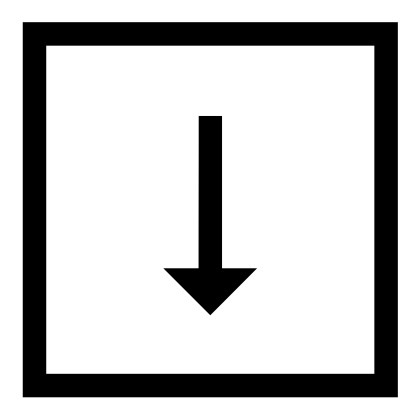	[7]
Cel/PLA	[Bmim][BF_4_]	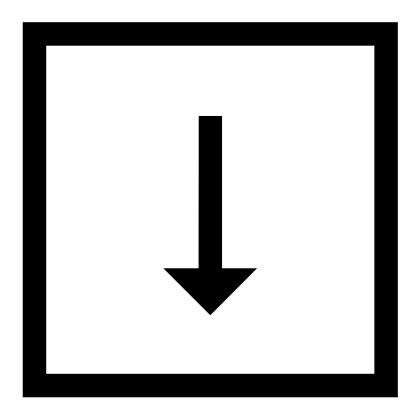	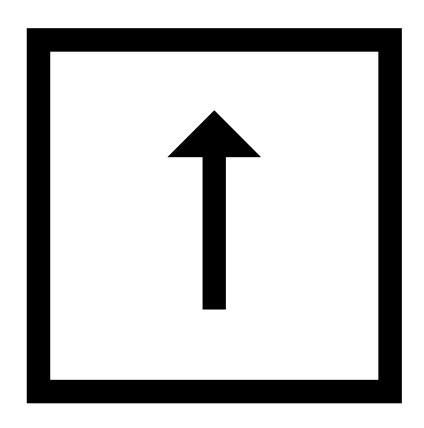	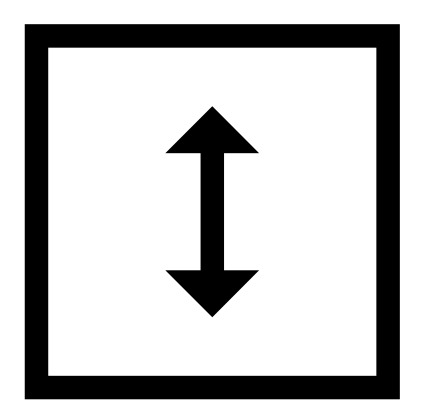	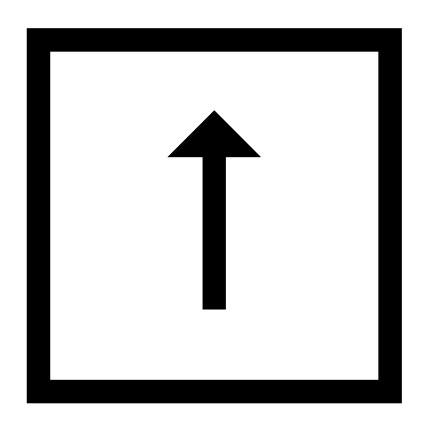	n/a	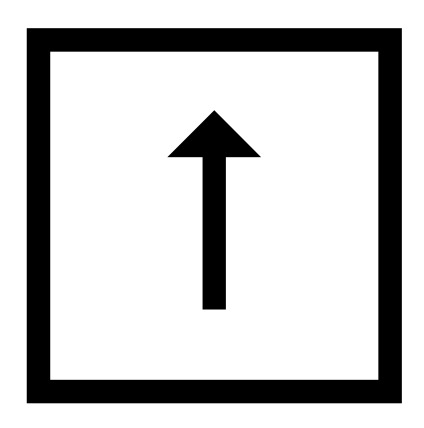	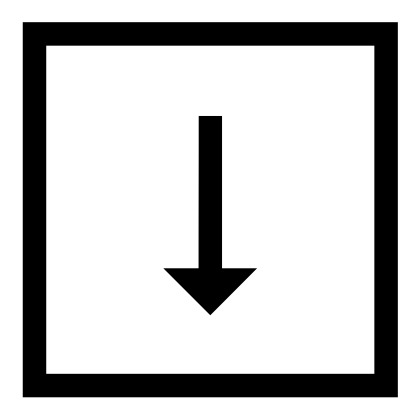	n/a	[3]
RS/PBS	[Dmim][NTf_2_]	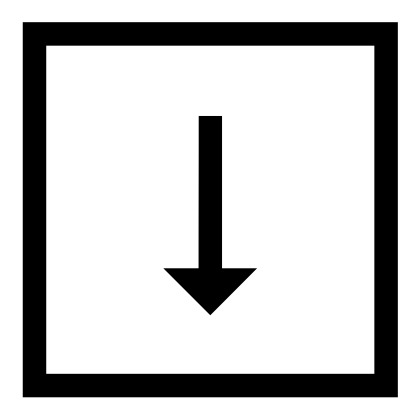	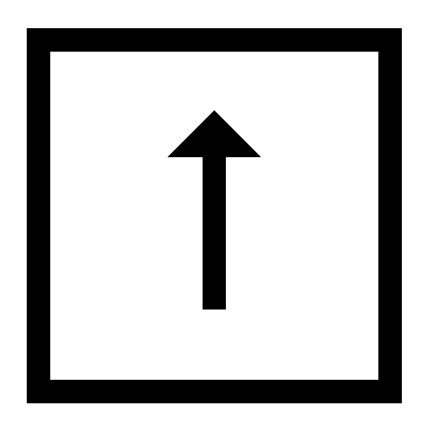	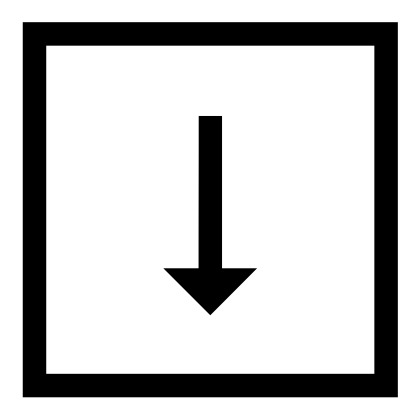	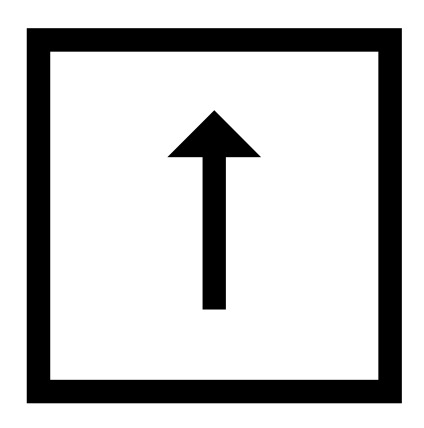	n/a	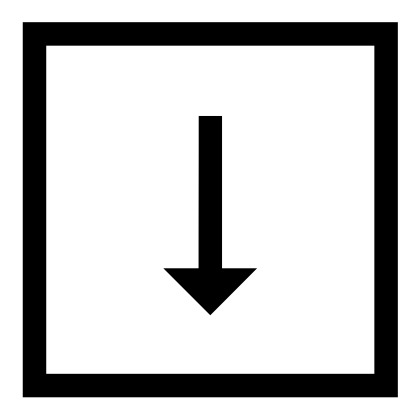	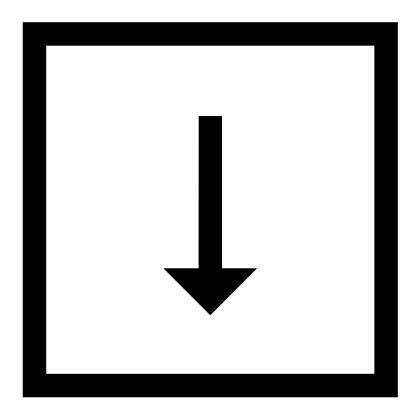	n/a	[10]
Chi/PLA	[Emim][OAc]	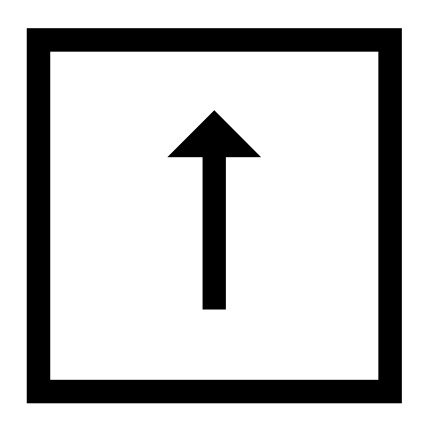	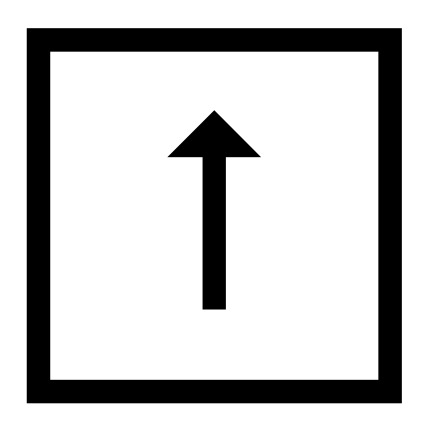	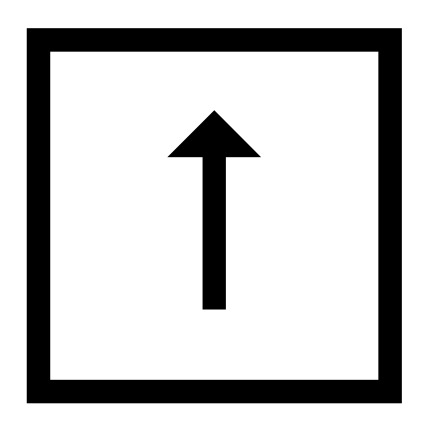	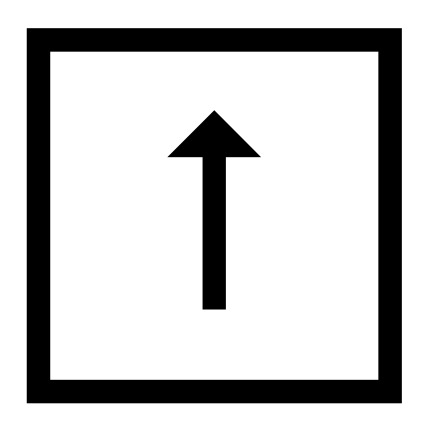	n/a	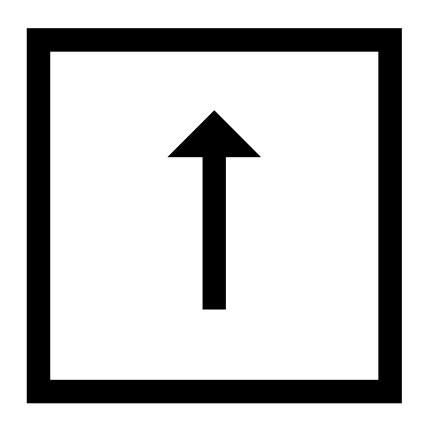	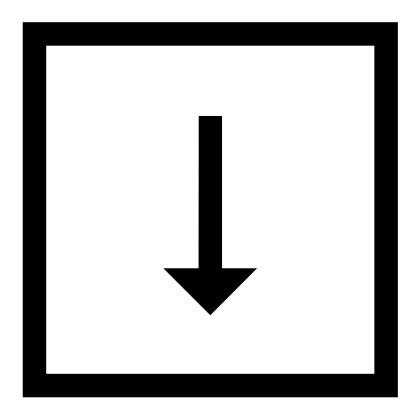	n/a	[11]

TS = tensile strength, EB = elongation at break, YM = Young’s modulus, *T*_d_ = decomposition temperature, *T*_g_ = glass transition temperature, *T*_m_ = melting temperature, FTIR = Fourier transform infrared, and XRD = X-ray diffraction. The symbol ‘
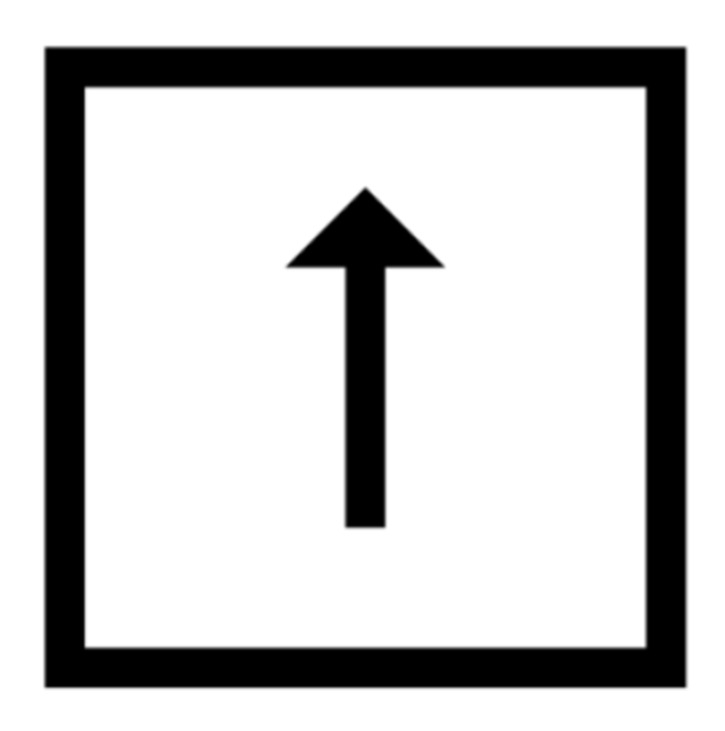
’ corresponds to an increase in the properties, and ‘
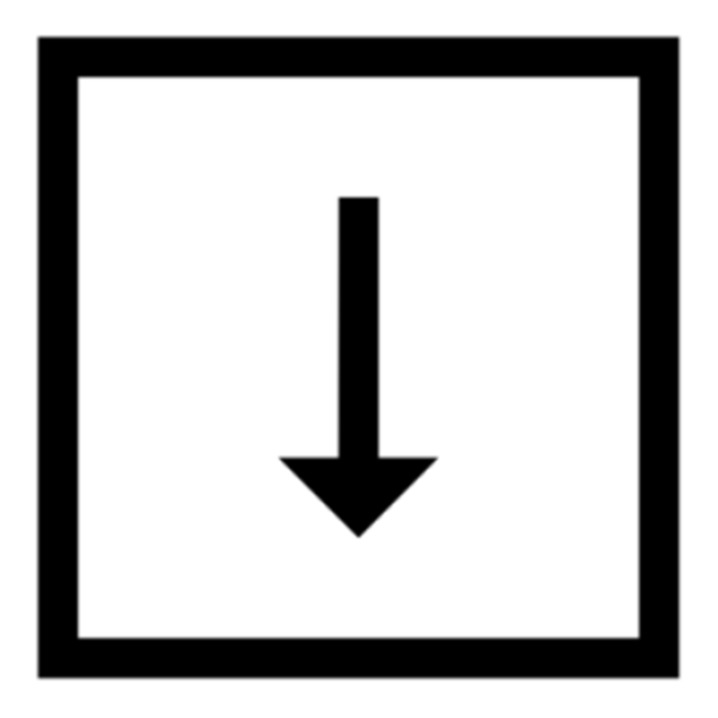
’ corresponds to a decrease in the properties, while ‘
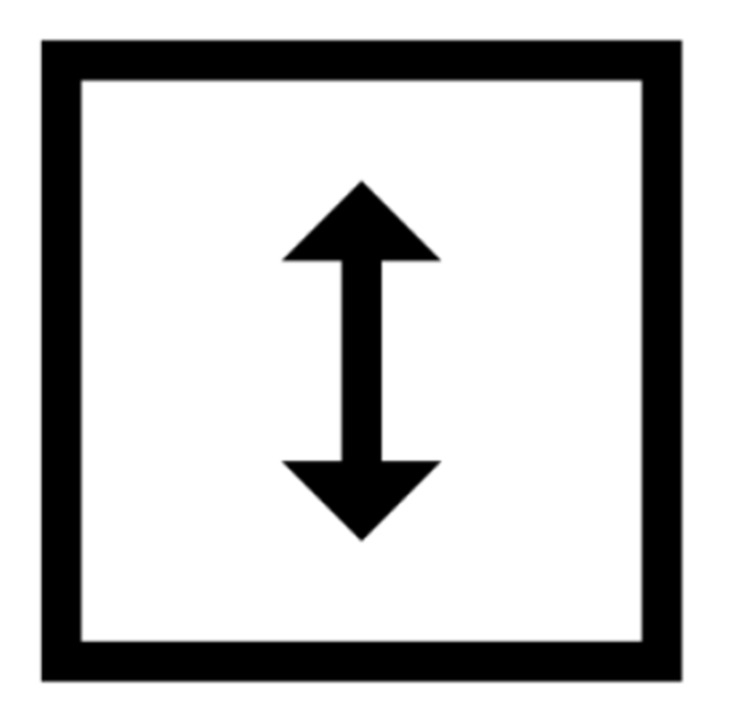
’ and ‘n/a’ describe unchanged and not available, respectively.

**Table 7:** Mechanical, thermal, and chemical properties of biodegradable polymer composites influenced by alkylphosphonium-based ionic liquids.

Biodegradable Polymer Composite	Alkylphosphonium-Based Ionic Liquid	Mechanical			Thermal			Chemical		References
		TS	EB	YM	*T*_d_	*T*_g_	*T*_m_	FTIR	XRD	
MMT/PBS	[P_6,6,6,14_][Cl]	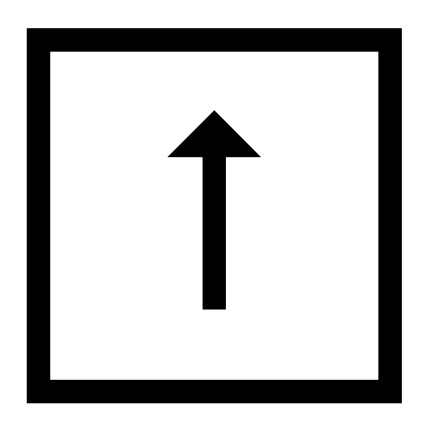	n/a	n/a	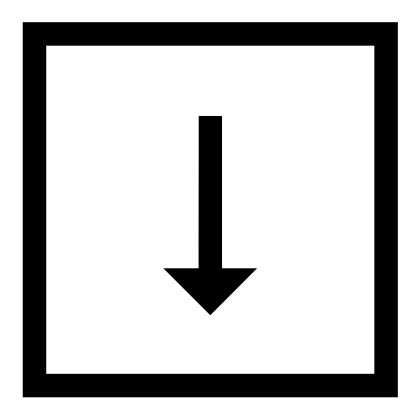	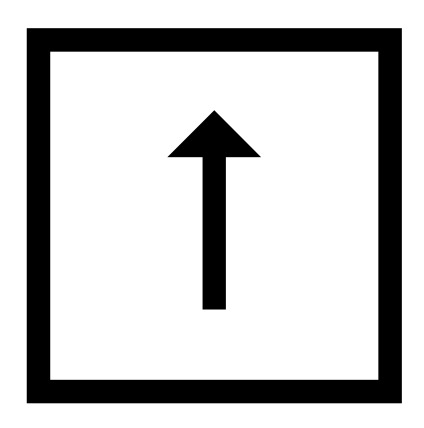	n/a	n/a	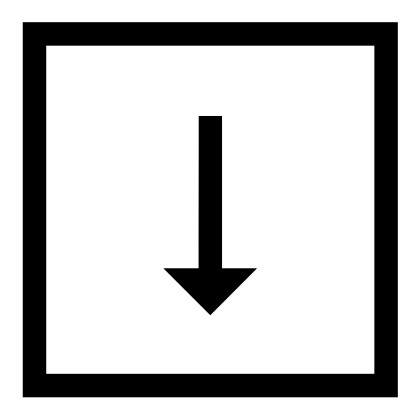	[28]
LDH/PCL	[P_6,6,6,14_][C_9_H_19_CO_2_]	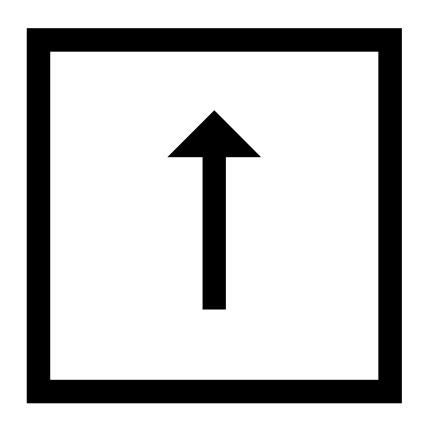	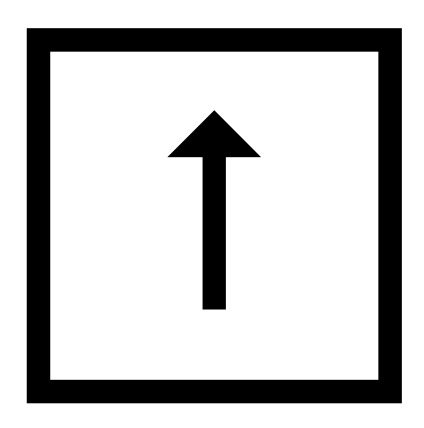	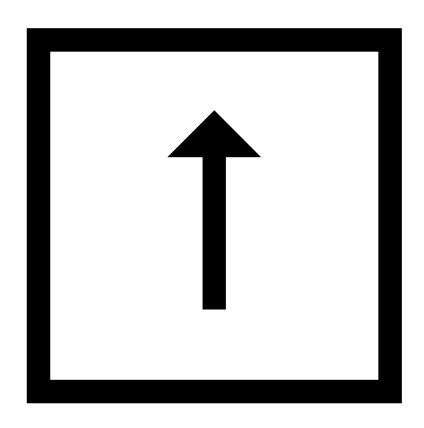	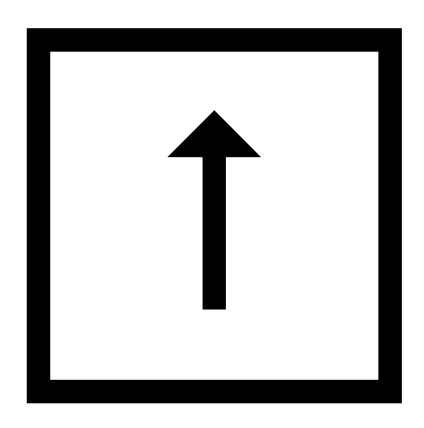	n/a	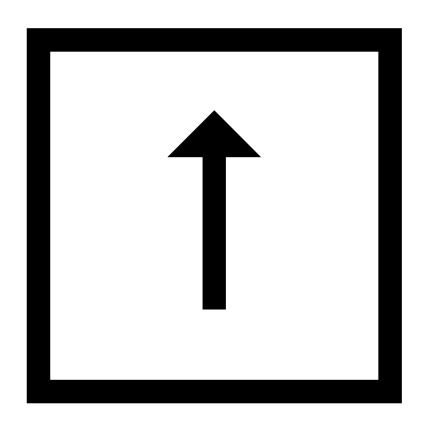	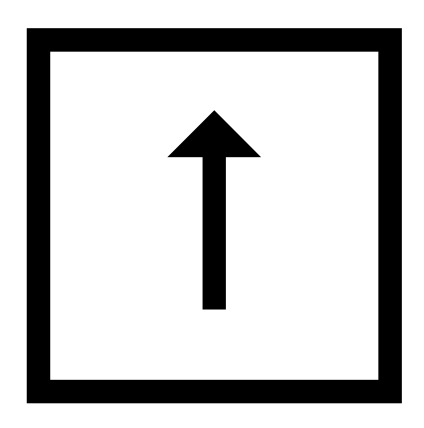	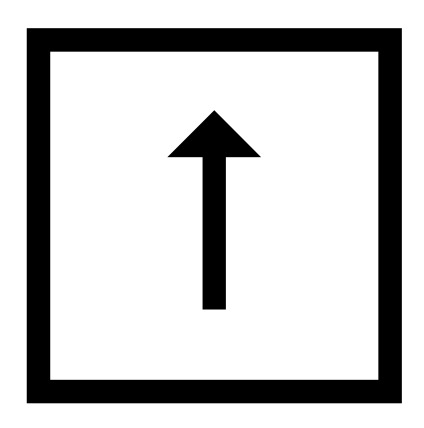	[26]
APP/PLA	[P_4,4,4,4_][BF_4_]	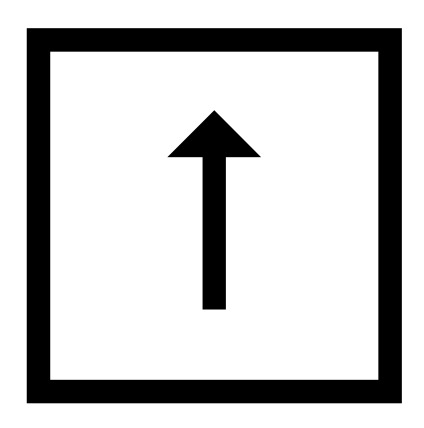	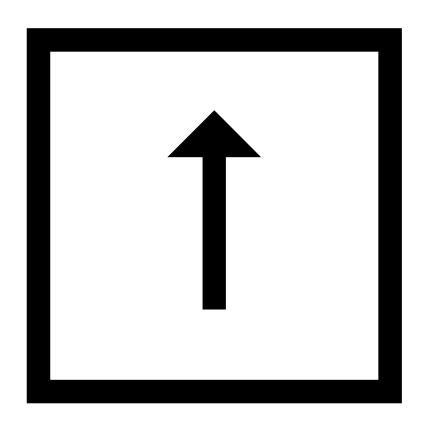	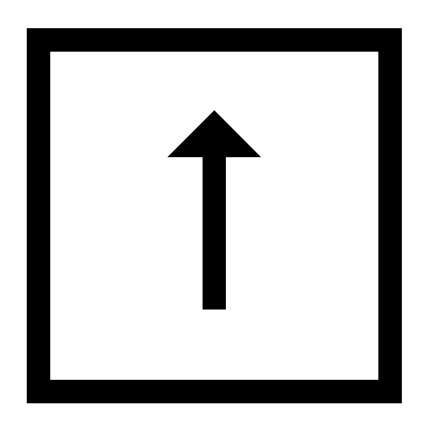	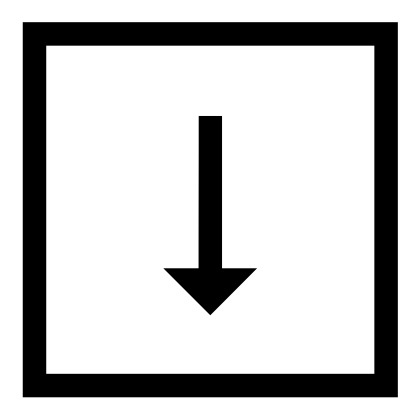	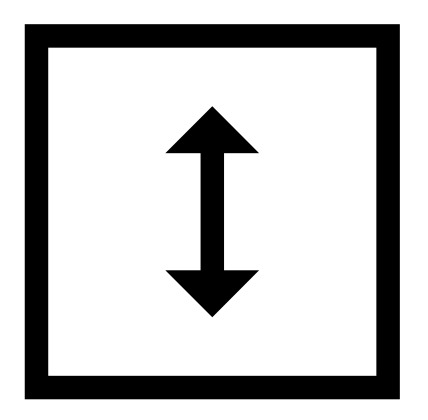	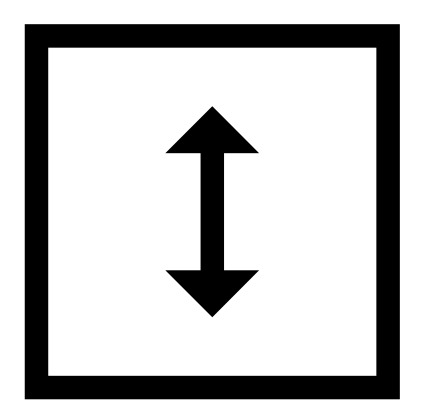	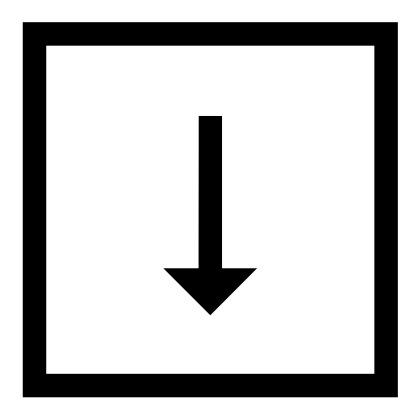	n/a	[30]
ZnO/PCL	[P_6,6,6,14_][C_9_H_19_CO_2_]	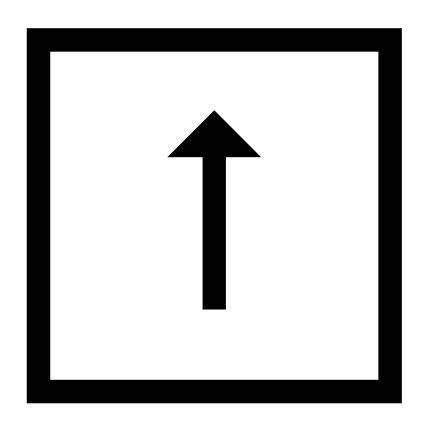	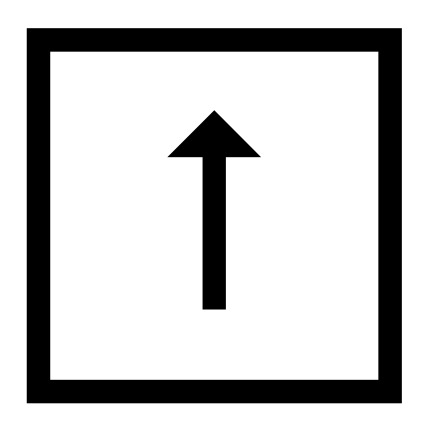	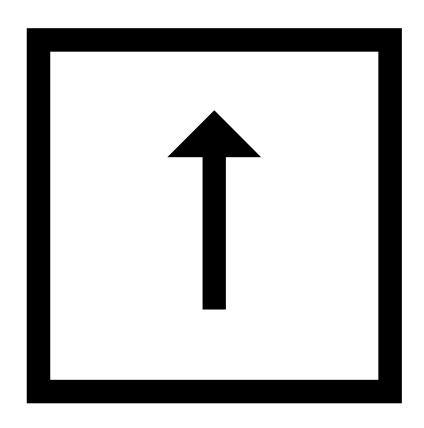	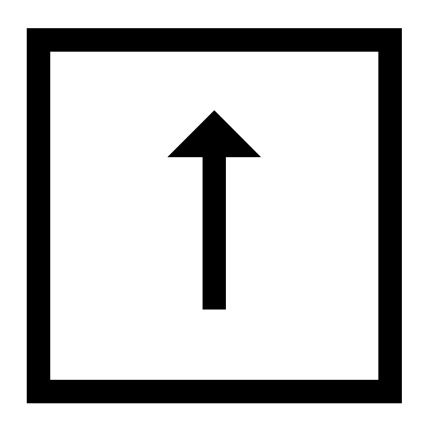	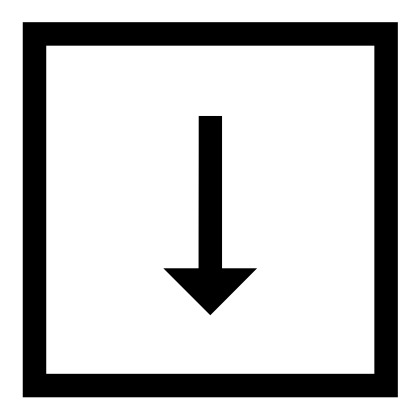	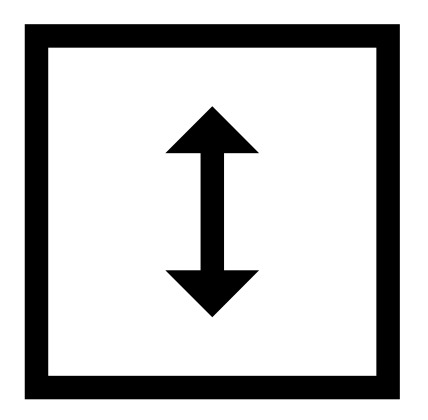	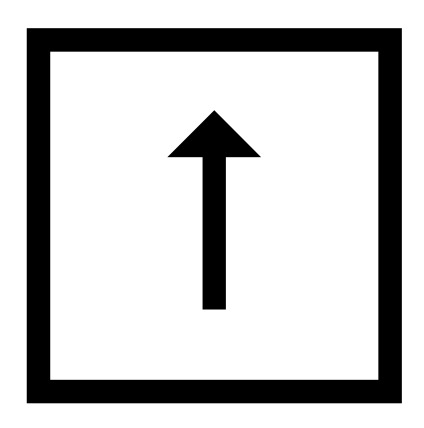	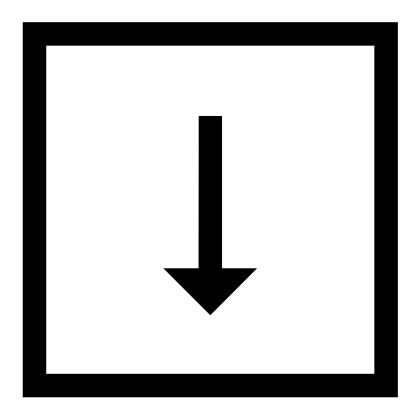	[31]

TS = tensile strength, EB = elongation at break, YM = Young’s modulus, *T*_d_ = decomposition temperature, *T*_g_ = glass transition temperature, *T*_m_ = melting temperature, FTIR = Fourier transform infrared, and XRD = X-ray diffraction. The symbol ‘
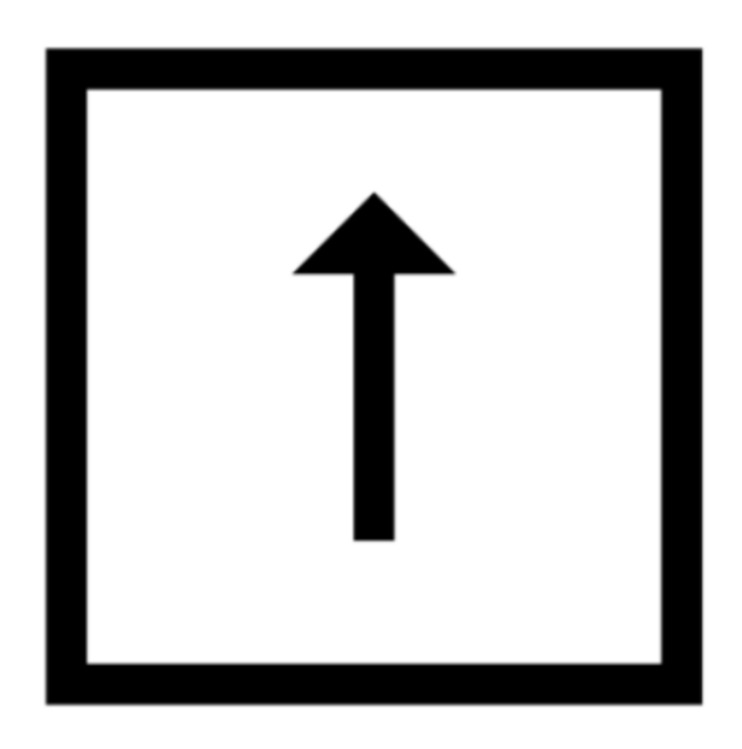
’ corresponds to an increase in the properties, and ‘
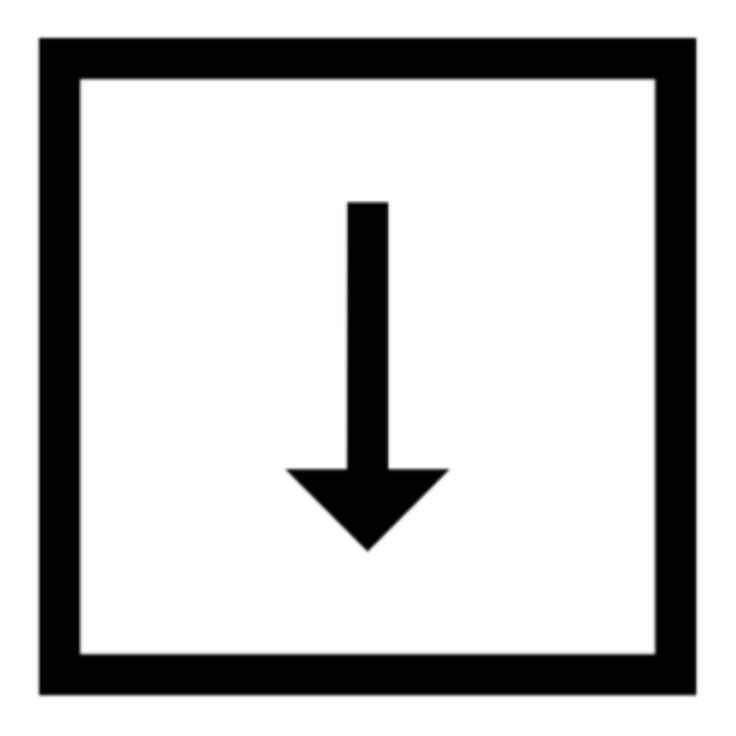
’ corresponds to a decrease in the properties, while ‘
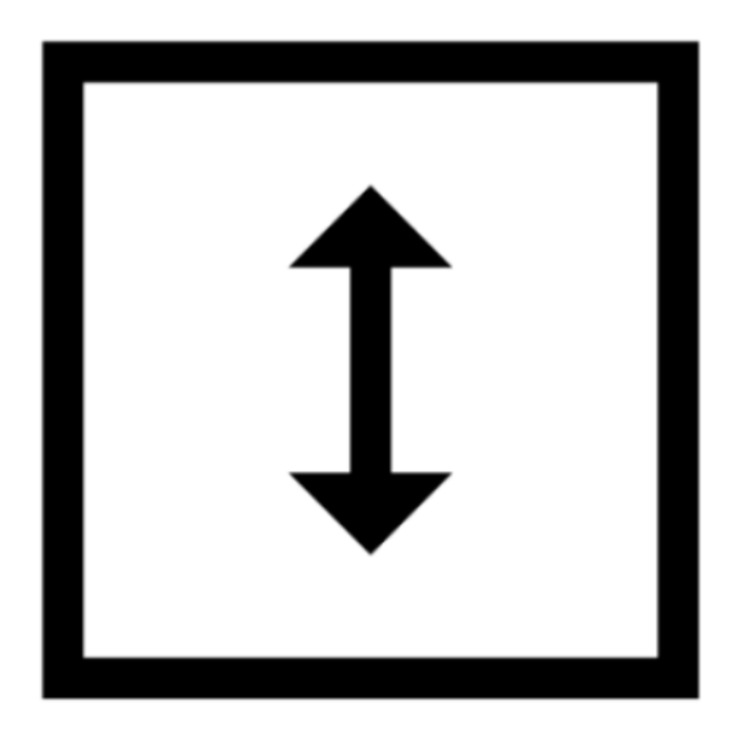
’ and ‘n/a’ describe unchanged and not available, respectively.

## Data Availability

Not applicable.

## References

[1] Zimina A., Senatov F., Choudhary R., Kolesnikov E., Anisimova N., Kiselevskiy M., Orlova P., Strukova N. (2020). Biocompatibility and Physico-Chemical Properties of Highly Porous PLA/HA Scaffolds for Bone Reconstruction. Polymers.

[2] Huang Q., Huang J., Chang P.R. (2014). Polycaprolactone grafting of cellulose nanocrystals in ionic liquid [BMIM]Cl. Wuhan Univ. J. Nat. Sci..

[3] Song X., Zhou L., Ding B., Cui X., Duan Y., Zhang J. (2018). Simultaneous improvement of thermal stability and redispersibility of cellulose nanocrystals by using ionic liquids. Carbohydr. Polym..

[4] Niu X., Huan S., Li H., Pan H., Rojas O.J. (2021). Transparent films by ionic liquid welding of cellulose nanofibers and polylactide: Enhanced biodegradability in marine environments. J. Hazard. Mater..

[5] Müller K., Zollfrank C. (2020). Ionic liquid aided solution-precipitation method to prepare polymer blends from cellulose with polyesters or polyamide. Eur. Polym. J..

[6] Xu A., Wang Y., Gao J., Wang J. (2019). Facile fabrication of a homogeneous cellulose/polylactic acid composite fi lm with improved biocompatibility, biodegradability and mechanical. Green Chem..

[7] Xu J., Chen Y., Tian Y., Yang Z., Zhao Z., Du W., Zhang X. (2021). Effect of ionic liquid 1-buyl-3-methylimidazolium halide on the structure and tensile property of PBS/corn starch blends. Int. J. Biol. Macromol..

[8] Zhao Z., Lei B., Du W., Yang Z., Tao D., Tian Y., Xu J., Zhang X. (2020). The effects of different inorganic salts on the structure and properties of ionic liquid plasticized starch/poly(butylene succinate) blends. RSC Adv..

[9] Liu D., Qi Z., Zhang Y., Xu J., Guo B. (2015). Poly (butylene succinate) (PBS)/ionic liquid plasticized starch blends: Preparation, characterization, and properties. Starch/Stärke.

[10] Shamsuri A.A., Md. Jamil S.N.A. (2020). Compatibilization Effect of Ionic Liquid-Based Surfactants on Physicochemical Properties of PBS/Rice Starch Blends: An Initial Study. Materials.

[11] Shamshina J.L., Zavgorodnya O., Berton P., Chhotaray P.K., Choudhary H., Rogers R.D. (2018). Ionic Liquid Platform for Spinning Composite Chitin-Poly(lactic acid) Fibers. ACS Sustain. Chem. Eng..

[12] Chakravarty J., Rabbi M.F., Chalivendra V., Ferreira T., Brigham C.J. (2020). Mechanical and biological properties of chitin/polylactide (PLA)/hydroxyapatite (HAP) composites cast using ionic liquid solutions. Int. J. Biol. Macromol..

[13] Islam S., Ahmed-haras M.R., Kao N., Gupta R., Husk R. (2019). Physico-mechanical properties of bio-composites fabricated from polylactic acid and rice husk treated with alkali and ionic liquid. Res. Commun. Eng. Sci. Technol..

[14] Quitadamo A., Massardier V., Valente M. (2018). Interactions between PLA, PE and wood flour: Effects of compatibilizing agents and ionic liquids. Holzforschung.

[15] Shamsuri A.A., Abdan K., Kaneko T. (2021). A Concise Review on the Physicochemical Properties of Biopolymer Blends Prepared in Ionic Liquids. Molecules.

[16] Wang H., Ren P.G., Liu C.Y., Xu L., Li Z.M. (2014). Enhanced toughness and strength of conductive cellulose-poly(butylene succinate) films filled with multiwalled carbon nanotubes. Cellulose.

[17] Lee H.H., Shin U.S., Jin G.Z., Kim H.W. (2011). Highly homogeneous carbon nanotube-polycaprolactone composites with various and controllable concentrations of ionically-modified-MWCNTs. Bull. Korean Chem. Soc..

[18] Wang P., Zhou Y., Hu X., Wang F., Chen J., Xu P., Ding Y. (2020). Improved mechanical and dielectric properties of PLA/EMA-GMA nanocomposites based on ionic liquids and MWCNTs. Compos. Sci. Technol..

[19] Lopes Pereira E.C., da Silva M.E.C.F., Pontes K., Soares B.G. (2019). Influence of Protonic Ionic Liquid on the Dispersion of Carbon Nanotube in PLA/EVA Blends and Blend Compatibilization. Front. Mater..

[20] Yan C.Y., Ren P.G., Zhang Z.P., Wang H., Li Z.M. (2015). In-situ preparation and characterization of highly oriented graphene oxide/cellulose-poly(butylene succinate) ternary composite films. Cellulose.

[21] Sánchez-rodríguez C., Avilés M.D., Pamies R., Carrión-vilches F.J., Sanes J., Bermúdez M.D. (2021). Extruded pla nanocomposites modified by graphene oxide and ionic liquid. Polymers.

[22] Fu Y., Liu L., Zhang J., Hiscox W.C. (2014). Functionalized graphenes with polymer toughener as novel interface modifier for property-tailored polylactic acid/graphene nanocomposites. Polymer.

[23] Xu P., Cui Z., Ruan G., Ding Y. (2019). Enhanced Crystallization Kinetics of PLLA by Ethoxycarbonyl Ionic Liquid Modified Graphene. Chin. J. Polym. Sci.

[24] Kredatusová J., Beneš H., Livi S., Pop-Georgievski O., Ecorchard P., Abbrent S., Pavlova E., Bogdał D. (2016). Influence of ionic liquid-modified LDH on microwave-assisted polymerization of ε-caprolactone. Polymer.

[25] Ha J.U., Xanthos M. (2010). Novel modifiers for layered double hydroxides and their effects on the properties of polylactic acid composites. Appl. Clay Sci..

[26] Bujok S., Hodan J., Beneš H. (2020). Effects of immobilized ionic liquid on properties of biodegradable polycaprolactone/ldh nanocomposites prepared by in situ polymerization and melt-blending techniques. Nanomaterials.

[27] Lins L.C., Bugatti V., Livi S., Gorrasi G. (2018). Ionic liquid as surfactant agent of hydrotalcite: Influence on the final properties of polycaprolactone matrix. Polymers.

[28] Tian Q., Qin S., Long L., Jiang Y., Zhou R., Zhou R., He W., Xu G. (2016). Effect of reaction media on clay dispersion and mechanical properties in poly(butylene succinate)/organoclay nanocomposites. J. Vinyl Addit. Technol..

[29] Arjona J.D.C., Silva-valenzuela M.G., Wang S., Valenzuela-diaz F.R. (2021). Biodegradable Nanocomposite Microcapsules for Controlled Release of Urea. Polymers.

[30] Jia Y.W., Zhao X., Fu T., Li D.F., Guo Y., Wang X.L., Wang Y.Z. (2020). Synergy effect between quaternary phosphonium ionic liquid and ammonium polyphosphate toward flame retardant PLA with improved toughness. Compos. Part B Eng..

[31] Bujok S., Peter J., Halecký M., Ecorchard P., Machálková A. (2021). Sustainable microwave synthesis of biodegradable active packaging films based on polycaprolactone and layered ZnO nanoparticles. Polym. Degrad. Stab. J..

[32] Su L., Castellano J., Sara D., Tcharkhtchi A., Ortega Z. (2021). Are Natural-Based Composites Sustainable?. Polymers.

[33] Amaro L., Correia D.M., Martins P.M., Botelho G., Carabineiro S.A.C., Ribeiro C., Lanceros-Mendez S. (2020). Morphology dependence degradation of electro-and magnetoactive poly(3-hydroxybutyrateco-hydroxyvalerate) for tissue engineering applications. Polymers.

[34] Aničić N., Kurtjak M., Jeverica S., Suvorov D., Vukomanović M. (2021). Antimicrobial Polymeric Composites with Embedded Nanotextured Magnesium Oxide. Polymers.

[35] Atmakuri A., Palevicius A., Kolli L., Vilkauskas A., Janusas G. (2021). Development and Analysis of Mechanical Properties of Caryota and Sisal Natural Fibers Reinforced Epoxy Hybrid Composites. Polymers.

[36] Sintharm P., Phisalaphong M. (2021). Green Natural Rubber Composites Reinforced with Black/White Rice Husk Ashes: Effects of Reinforcing Agent on Film’s Mechanical and Dielectric Properties. Polymers.

[37] Brounstein Z., Yeager C.M., Labouriau A. (2021). Development of Antimicrobial PLA Composites for Fused Filament Fabrication. Polymers.

[38] Adli S.A., Ali F., Azmi A.S., Anuar H., Nasir N.A.M., Hasham R., Idris M.K.H. (2020). Development of biodegradable cosmetic patch using a polylactic acid/phycocyanin-alginate composite. Polymers.

[39] Bi S., Barinelli V., Sobkowicz M.J. (2020). Degradable controlled release fertilizer composite prepared via extrusion: Fabrication, characterization, and release mechanisms. Polymers.

[40] Cao X., Tian N., Dong X., Cheng C. (2019). Polylactide Composite Pins Reinforced with Bioresorbable Continuous Glass Fibers Demonstrating Bone-like Apatite Formation and Spiral Delamination Degradation. Polymers.

[41] Shamsuri A.A., Daik R., Md. Jamil S.N.A. (2021). A Succinct Review on the PVDF/Imidazolium-Based Ionic Liquid Blends and Composites: Preparations, Properties, and Applications. Processes.

[42] Shamsuri A.A., Jamil S.N.A.M. (2021). Application of Quaternary Ammonium Compounds as Compatibilizers for Polymer Blends and Polymer Composites—A Concise Review. Appl. Sci..

[43] Momeni S., Ghomi E.R., Shakiba M., Shafiei-Navid S., Abdouss M., Bigham A., Khosravi F., Ahmadi Z., Faraji M., Abdouss H. (2021). The effect of poly (Ethylene glycol) emulation on the degradation of pla/starch composites. Polymers.

[44] Puchalski M., Szparaga G., Biela T., Gutowska A. (2018). Molecular and Supramolecular Changes in Polybutylene Succinate (PBS) and Polybutylene Succinate Adipate (PBSA) Copolymer during Degradation in Various Environmental Conditions Michał. Polymers.

[45] Coiai S., Laura M., Lorenzo D., Cinelli P., Righetti M.C., Passaglia E. (2021). Binary Green Blends of Poly(lactic acid) with Poly(butylene adipate-co-butylene terephthalate) and Poly(butylene succinate-co-butylene adipate) and Their Nanocomposites. Polymers.

[46] Calabia B.P., Ninomiya F., Yagi H., Oishi A., Taguchi K., Kunioka M., Funabashi M. (2013). Biodegradable poly(butylene succinate) composites reinforced by cotton fiber with silane coupling agent. Polymers.

[47] Diaz C.A., Shah R.K., Evans T., Trabold T.A., Draper K. (2020). Thermoformed Containers Based on Starch and Starch/Coffee Waste Biochar Composites. Energies.

[48] Shamsuri A.A., Jamil S.N.A.M., Abdan K. (2021). Processes and Properties of Ionic Liquid-Modified Nanofiller/Polymer Nanocomposites—A Succinct Review. Processes.

[49] Livi S., Duchet-rumeau J., Pham T., Gérard J. (2010). A comparative study on different ionic liquids used as surfactants: Effect on thermal and mechanical properties of high-density polyethylene nanocomposites. J. Colloid Interface Sci..

[50] Tang Z., Huang J., Wu X., Guo B., Zhang L., Liu F. (2015). Interface Engineering toward Promoting Silanization by Ionic Liquid for High-Performance Rubber/Silica Composites. Ind. Eng. Chem. Res..

